# Analysis of treatment process time for real‐time‐image gated‐spot‐scanning proton‐beam therapy (RGPT) system

**DOI:** 10.1002/acm2.12804

**Published:** 2019-12-30

**Authors:** Takaaki Yoshimura, Shinichi Shimizu, Takayuki Hashimoto, Kentaro Nishioka, Norio Katoh, Tetsuya Inoue, Hiroshi Taguchi, Koichi Yasuda, Taeko Matsuura, Seishin Takao, Masaya Tamura, Yoichi M. Ito, Yuto Matsuo, Hiroshi Tamura, Kenji Horita, Kikuo Umegaki, Hiroki Shirato

**Affiliations:** ^1^ Proton Beam Therapy Center Hokkaido University Hospital Sapporo Japan; ^2^ Department of Radiation Oncology Faculty of Medicine Hokkaido University Sapporo Japan; ^3^ Global Station for Quantum Medical Science and Engineering Global Institution for Collaborative Research and Education (GI‐CoRE) Hokkaido University Sapporo Japan; ^4^ Department of Radiation Medicine Faculty of Medicine Hokkaido University Sapporo Japan; ^5^ Department of Radiation Oncology Hokkaido University Hospital Sapporo Japan; ^6^ Faculty of Engineering Hokkaido University Sapporo Japan; ^7^ Department of Statistical Data Science The Institute of Statistical Mathematics Tokyo Japan

**Keywords:** beam‐delivery efficiency, interplay effect, organ motion, spot‐scanning proton‐beam therapy, treatment time

## Abstract

We developed a synchrotron‐based real‐time‐image gated‐spot‐scanning proton‐beam therapy (RGPT) system and utilized it to clinically operate on moving tumors in the liver, pancreas, lung, and prostate. When the spot‐scanning technique is linked to gating, the beam delivery time with gating can increase, compared to that without gating. We aim to clarify whether the total treatment process can be performed within approximately 30 min (the general time per session in several proton therapy facilities), even for gated‐spot‐scanning proton‐beam delivery with implanted fiducial markers. Data from 152 patients, corresponding to 201 treatment plans and 3577 sessions executed from October 2016 to June 2018, were included in this study. To estimate the treatment process time, we utilized data from proton beam delivery logs during the treatment for each patient. We retrieved data, such as the disease site, total target volume, field size at the isocenter, and the number of layers and spots for each field, from the treatment plans. We quantitatively analyzed the treatment process, which includes the patient load (or setup), bone matching, marker matching, beam delivery, patient unload, and equipment setup, using the data obtained from the log data. Among all the cases, 90 patients used the RGPT system (liver: n = 34; pancreas: n = 5; lung: n = 4; and prostate: n = 47). The mean and standard deviation (SD) of the total treatment process time for the RGPT system was 30.3 ± 7.4 min, while it was 25.9 ± 7.5 min for those without gating treatment, excluding craniospinal irradiation (CSI; head and neck: n = 16, pediatric: n = 31, others: n = 15); for CSI (n = 11) with two or three isocenters, the process time was 59.9 ± 13.9 min. Our results demonstrate that spot‐scanning proton therapy with a gating function can be achieved in approximately 30‐min time slots.

## 1 INTRODUCTION

The number of proton‐beam therapy facilities is rapidly increasing worldwide.^1^ Among the several treatment delivery systems in proton‐beam therapy, spot‐scanning proton therapy (SSPT) is one of the more promising technologies. In spot‐scanning methods, the prescribed dose for each field is delivered by thousands or tens of thousands of pencil beams, spot‐by‐spot and layer‐by‐layer, from the nozzle to the target.^2^ This method has a higher beam utilization than conventional methods such as the double scatter method. Furthermore, it does not require boluses or collimators and enables intensity‐modulated proton therapy (IMPT).^3^ Hence, this scanning method has become the mainstay in many new facilities.

However, SSPT is sensitive to the large uncertainty in the dose distribution due to the interplay effects between the time‐dependent scanning beam delivery and the tumor motion.^4^ For example, when the interplay effect, such as baseline shift or drift, occurs, it causes a hot or cold spot in the target. To solve these problems, rescanning, respiratory gating, and tumor tracking using implanted fiducial markers can be applied.^5^, ^6^, ^7^, ^8^ These tools for motion management during radiotherapy are effective. However, the beam delivery time is longer, compared to the other approaches. As skin motion and internal tumor motion do not always correlate well with each other, it is essential to identify the tumor itself from the fiducial marker implanted adjacent to the tumor. Based on these considerations, we developed and clinically operated a real‐time‐image gated‐spot‐scanning proton‐beam therapy (RGPT) system without using the respiration waveform inherited from the basic properties of the x ray real‐time tumor‐tracking radiation therapy (RTRT) system developed by Shirato et al.^4^, ^9^, ^10^, ^11^ Furthermore, the feasibility of synchrotron‐based SSPT with a gating function was demonstrated for clinical purposes.

It is important for proton therapy facilities to consider the treatment‐room throughput and efficiency. In previous studies, Suzuki et al. evaluated the treatment time for passive scattering proton therapy and SSPT in a multiroom facility.^12^, ^13^ Using the gating function, we can use a proton pencil beam to irradiate a moving tumor with high accuracy, but the treatment‐room throughput may be reduced because of the longer beam delivery time compared to that without the gating function.

At the SSPT facility, the daily treatment schedule for a patient is divided into fixed 30‐min time slots.^13^ For pediatric patients who require anesthesia, many proton facilities generally allot a fixed time slot of 30–90 min for a treatment session depending upon the patient characteristics. Farace et al.^14^ demonstrated that the average total in‐room time for craniospinal irradiation (CSI) pediatric patients was 80 min, under anesthesia, and 67 min without anesthesia, which included 32 min for beam delivery. Therefore, for efficient treatment‐room operation, it is important to complete the treatment within 30 min, irrespective of beam delivery with or without gating.

A previous study by Vinogradskiy et al. demonstrated the treatment time, with the real‐time tumor‐tracking method for pancreatic stereotactic body radiation therapy (SBRT).^15^ However, to the best of our knowledge, the treatment process time for synchrotron‐based gated proton‐beam therapy has not been analyzed. The results of our study would be beneficial for many proton therapy facilities planning to introduce marker‐based gated proton‐beam therapy. In this study, we quantitatively analyzed the treatment process time using the data from the machine log files in our facility.

## 2 MATERIALS AND METHODS

### 2.1 Patient data

The quantitative analysis performed in this study includes 152 patients, who had previously received SSPT at our institution from October 2016 to June 2018. We categorized patients based on the disease site as liver, pancreas, lung, prostate, head and neck, pediatric, CSI, and others. The number of patients for each category is listed in Table 1. Moreover, we used the anesthesia machine for pediatric patients, who needed anesthesia. This study is approved by the ethics committee of our hospital (016‐0454).

**Table 1:** Characteristics of the patients in this study. Ten of 11 craniospinal irradiation (CSI) patients were pediatric. Two pediatric CSI patients were treated under general anesthesia.

	n	(%)	Age	
			Mean	Range
Number of Patients	152		55.5	0‐88
Sex				
Male	106	69.7%		
Female	46	30.3%		
Categories				
With gating				
Prostate	47	30.9%		
Liver	34	22.4%		
Pancreas	5	3.3%		
Lung	4	2.6%		
Without gating				
Head & Neck	16	10.5%		
Pediatric	21	13.8%		
Pediatric CSI	8	5.3%		
Pediatric CSI with general anesthesia	2	1.3%		
Others	14	9.2%		
Adult CSI	1	0.7%		

### 2.2 Synchrotron‐based real‐time‐image gated‐spot‐scanning proton‐beam therapy (RGPT) system

We used the gating function to manage the internal motions of the liver, pancreas, lung, and prostate tumors. In the RGPT system, we used a 1.5‐mm or 2.0‐mm diameter gold internal fiducial marker near the tumor, before the treatment planning computed tomography (CT) scan. The reference marker position was determined by the treatment planning CT, which was mainly taken with exhalation. No training or visual monitoring of breath was used during the treatment planning CT and the treatment was performed with free and natural breathing.^16^ To gate the proton beam in this system, we checked the location of the marker using two orthogonal sets of x ray fluoroscopes during radiotherapy using real‐time pattern recognition technology. This was applied for the automatic recognition of the projected figure of the gold marker in fluoroscopic images. The details of the inserted fiducial marker are shown in a previous report.^17^ The pulse rate for fluoroscopy was set to 30 or 15 Hz for liver, pancreas, and lung patients, whereas it was set to 1 Hz for prostate patients.

In the RGPT system, the proton beam is gated when the marker enters a preassigned gating window. The gating window tolerance in the actual treatment for each patient was set to ±2.0 mm, based on a previous study, where the RGPT yielded good dose distributions with a ±2.0‐mm gating window.^18^, ^19^ Because the gold internal fiducial marker is monitored through fluoroscopy, we can monitor not only the internal movement due to breathing but also the peristaltic movement of the intestinal tract, and gate irradiation is clinically performed with an accuracy of ±2.0 mm.

**Figure 1:**
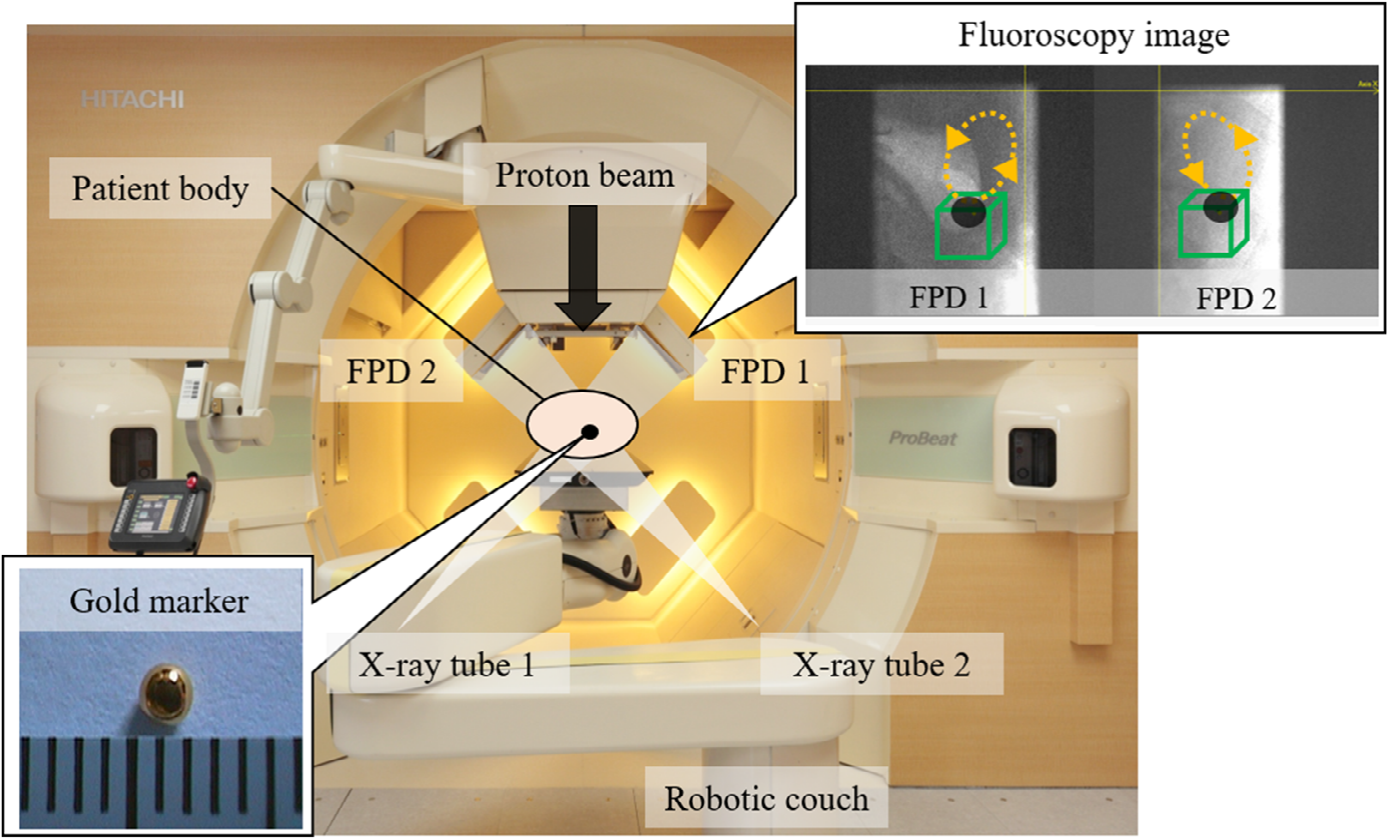
Overview of the real‐time‐image gated‐spot‐scanning proton‐beam therapy system in our facility. In the fluoroscopy images obtained from two sets of flat panel detectors (FPD 1 and FPD 2), the dotted lines indicate the trajectory of the marker in the patient body and box indicates the ± 2.0‐mm gating window from the marker coordinates in the treatment plan.

We installed a multiple gating beam delivery function as part of the proton‐beam control to improve the irradiation efficiency of the gating technique. Details on multiple gating beam delivery for synchrotron operation are mentioned in a previous study.^20^ As the synchrotron operates, this function improves the gate irradiation efficiency and reduces $T_{\mathrm{BSR}}\left(X,V,R\right)$. Figure 1 presents an overview of the RGPT system.

### 2.3 Treatment planning

All the patients received a planning CT scan with a slice thickness of 1.25‐ or 2.5‐mm. Treatment planning for all the patients was performed using the VQA treatment planning system (TPS; Hitachi, Ltd., Tokyo, Japan) with single‐field uniform dose (SFUD) proton treatment or IMPT with robust optimization. In the treatment plan, the dose was prescribed to the clinical target volume (CTV). In our facility, the beam‐specific optimization margins for the CTV are specified.^21^, ^22^ A treatment plan was prepared for each patient, and if necessary, a replan or boost plan was devised. The number of treatment plans for each category is listed in Table 2.

**Table 2:** Characteristics of the treatment plans in this study.

Categories	Number of treatment plans	Number of sessions	Number of fields per session		Optimization methods			CTV (mL)	
			Median	Min–Max	SFUD	IMPT	IMPT + SFUD	Mean	Range
Prostate	48	1280	4	2–4	39	9	0	67.2	31.4–126.4
Liver	40	610	2	2–4	39	1	0	288.8	1.7–2246.2
Pancreas	6	125	2	2–2	6	0	0	220.3	38.6–382.3
Lung	4	40	3	3–3	4	0	0	39.5	4.9–77.6
Head & neck	27	439	3	2–3	3	24	0	230.9	21.2–626.8
Pediatric	45	600	2	1–5	17	25	3	262.8	14.1–1423.1
CSI	11	156	4	3–4	0	11	0	1602.0	1380.3–1897.6
Others	20	327	2	1–3	0	0	0	291.3	1.5–878.5
Total	201	3577			108	70	3		

Categories	Field size [cross line (mm)]		Field size [in line (mm)]		Number of layers per session		Number of spots per session	
	Mean	Range	Mean	Range	Mean	Range	Mean	Range
Prostate	66.4	44.8–93.7	65.8	43.8–93.7	80	44–113	4447	2789–6810
Liver	83.7	39.9–196.0	81.2	39.9–217.0	67	25–153	10 602	2297–49 950
Pancreas	115.8	75.5–134.0	106.3	97.1–117.5	63	43–73	10 146	4638–12 216
Lung	61.6	33.0–102.0	47.0	27.7–66.0	94	67–141	7120	2803–11 157
Head & neck	116.5	43.0–245.0	129.3	56.6–240.0	109	32–210	11 418	2957–26 562
Pediatric	97.7	36.0–225.0	101.6	30.2–308.0	107	27–237	9157	2106–26 869
CSI	128.7	56.0–189.0	283.0	182.0–396.0	211	174–229	59 605	40 913–72 362
Others	97.2	30.0–157.0	114.6	30.0–203.0	79	24–137	10 729	2095–24 442

Abbreviations: CTV, clinical target volume; IMPT, intensity‐modulated proton therapy; SFU; single‐field uniform.

### 2.4 Treatment process

We used the synchrotron‐based spot‐scanning proton‐beam system, PROBEAT‐RT (Hitachi, Ltd., Tokyo, Japan), in our facility to deliver treatments. The synchrotron beam has a maximum range of 30 g/cm^2^ and an irradiation field size of 30 cm × 40 cm. The details of our synchrotron‐based spot‐scanning proton‐beam delivery system with the gating function were provided in a previous report.^10^ We used the TPS to calculate the proton dose and the electronic medical record system MOSAIQ (Elekta Software, Sunnyvale, CA) to record the pertinent patient, treatment, and machine parameters.

Bone matching for the patient setup was performed by comparing the digitally reconstructed radiographs computed by the TPS with the digital radiographs (DRs) produced by the orthogonal kV x ray imaging systems. Moreover, orthogonal DRs for each isocenter were obtained for a multi‐isocenter plan, as in the CSI for a pediatric patient.

When using the gating function, bone matching was performed in a similar manner to that without gating. After bone matching, the gantry was rotated from the setup angle to the treatment angle, and marker matching was performed using fluoroscopy. The gate signal was only activated when the internal marker was within the gating window. In gated irradiation, the interfractional baseline shift or drift should be considered.^23^, ^24^ If a baseline shift or drift occurred during treatment, as in the case where it is necessary to move the couch during irradiation, proton‐beam irradiation was temporarily stopped, and the couch was moved as per the amount calculated by marker matching. In such a case, the physician permits the couch to be moved within the shift tolerance for robust evaluation. Although this tolerance for each direction is basically set at 8 mm at the maximum for liver and 5 mm at the maximum for others in our institution, often stricter tolerances are required. Physicians determined the final tolerance range for a specific patient based on the robust evaluation. The frequency corresponding to the baseline shift or drift was calculated as the ratio of the number of sessions, which required baseline shift or drift, among all the sessions.

**Figure 2:**
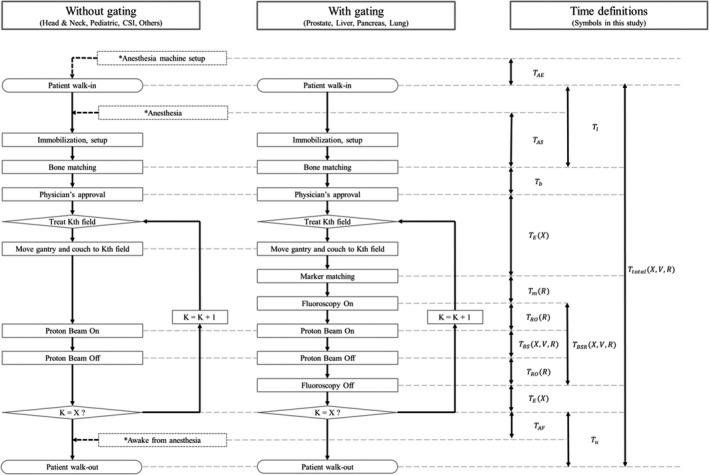
Difference in the treatment process flow for a spot‐scanning proton therapy treatment session in our facility, which has X treatment fields without and with the gating function. K is the index number of the field. The treatment process related to anesthesia for pediatric CSI patients are indicated by dotted lines. The corresponding definitions, listed in Table 3, are indicated on the right.

In this study, we used the machine log data to analyze the patient treatment process flow. The treatment process time without gating has already been defined in previous studies.^12^, ^13^ However, in order to include the gating function as the treatment process time, we redefined the treatment process time up to the marker gated irradiation, as presented in Table 3. Figure 2 depicts the differences in the treatment process in a session with and without the gating function.

**Table 3:** Definition of the symbols and terms used in this paper.

Symbol	Definition	Defined in previous studies^13^
*X*	Number of fields per session	○
*V*	CTV in cubic centimeters	○
$T_{\mathrm{total}}\left(X,\;V\right)$	Total treatment time per session without the use of the gating function	○
$T_{\mathrm{BS}}\left(X,V\right)$	Beam delivery time per session without the use of the gating function	○
$T_{X}\left(X\right)$	Number of field‐related treatment process times per session without using the gating function	○
*R*	Usage of the gating function (with RGPT system: *R* = 1, without RGPT system: *R* = 0)	
$T_{\mathrm{total}}\left(X,\;V,\;R\right)$	Total treatment time per session for image gated proton‐beam therapy	
$T_{walk-in}$	Logged time of patient walk‐in to the treatment room	
$T_{walk-out}$	Logged time of patient walk‐out from the treatment room	
$T_{\mathrm{BSR}}\left(X,V,R\right)$	Beam delivery time per session for image gated proton‐beam therapy, including the gating operation time	
$T_{X}\left(X,R\right)$	Number of field‐related treatment process times per session for image gated proton‐beam therapy	
$T_{E}\left(X\right)$	Equipment‐related time per session for image gated proton‐beam therapy, including the time for gantry rotation and couch movement	○
$T_{p}\left(R\right)$	Patient‐related time per session for image gated proton‐beam therapy, including the time for patient loading, immobilization, setup, bone matching, marker matching, and unloading	
*T*_1_	Patient loading time, including the time for patient immobilization from patient walk‐in to the treatment room	
*T_b_*	Bone matching time per session	
$T_{m}\left(R\right)$	Marker matching time per session	
*T_u_*	Patient unloading time from the completion of irradiation to patient walk‐out from the treatment room	
$T_{\mathrm{BS}}\left(X,V,R\right)$	Beam delivery time per session for image gated proton‐beam therapy	
$T_{\mathrm{RO}}\left(R\right)$	Gating operation time per session. This time is required for exchanging signals between devices	
*T_AE_*	Process time to prepare the general anesthesia machine	
*T_AS_*	Process time required from the administration of anesthesia to the start of bone matching	
*T_AF_*	Process time required from the completion of irradiation to awakening	○

We analyzed the treatment process time for one session, as shown in Fig. 2. When the patient walked into the treatment room, the therapists immobilized the patient on the couch using appropriate immobilization devices. If general anesthesia was needed, sedation was performed using an anesthesia machine prepared in the treatment room. After the patient setup, the therapist performed bone matching; the physician then approved the patient setup. The therapist rotated the gantry from the setup angle to the irradiation angle of the first field. When the first field was completed, the therapist moved the gantry to the next irradiation angle. This treatment process was continued until all the planned irradiation fields were delivered. After the completion of all the irradiation fields, the patient was unloaded from the couch.

Suzuki et al.^13^ demonstrated that the treatment process time required for proton‐beam irradiation depends on the numbers of spots and layers, that is, the volume of the target, and that the total treatment process time also depends on the number of irradiation fields.

Suzuki et al. defined the total treatment process time ($T_{\mathrm{total}}\left(X,V\right)$) without the gating function; it is the sum of the beam delivery time ($T_{\mathrm{BS}}\left(X,V\right)$) and the number of field‐related treatment process times (*T_X_* (*X*))^12^, ^13^:

(1) $$T_{\mathrm{total}}\left(X,V\right)=T_{\mathrm{BS}}\left(X,V\right)+T_{X}\left(X\right),$$

where *X* is the number of fields per session and *V* is the CTV. To extend the definition of the treatment process for image gated proton‐beam therapy, as indicated in Table 2, we redefined the total treatment process time per session $\left(T_{\mathrm{total}}\left(X,V,R\right)\right)$, the beam delivery time per session ($T_{\mathrm{BSR}}\left(X,V,R\right)$), and the number of field‐related treatment process times ($T_{X}\left(X,R\right)$) as functions of *X*, *V*, and *R*. This is because the total treatment process time increases with the number of fields per session, the total target volume, and the use of the gating function. Here, *R* is an independent variable (with gating: *R* = 1 and without gating: *R* = 0).

In this study, we logged the time of patient walk‐in ($T_{walk-in}$) and walk‐out ($T_{walk-out}$) from the treatment room. Hence, we defined $T_{\mathrm{total}}\left(X,V,R\right)$ as the duration of each treatment session from patient walk‐in to walk‐out:

(2) $$T_{\mathrm{total}}\left(X,V,R\right)=T_{walk-out}-T_{walk-in}=T_{\mathrm{BSR}}\left(X,V,R\right)+T_{X}\left(X,R\right),$$

where $T_{\mathrm{BSR}}\left(X,V,R\right)$ is the beam delivery time per session, $T_{X}\left(X,R\right)$ is the number of field‐related treatment process times, $T_{walk-in}$ is the logged time of patient walk‐in to the treatment room, and $T_{walk-out}$ is the logged time of patient walk‐out from the treatment room. $T_{X}\left(X,R\right)$ is expressed as a linear function of the number of fields per session:

(3) $$T_{X}\left(X,R\right)=T_{E}\left(X\right)+T_{p}\left(R\right),$$

where $T_{E}\left(X\right)$ is the equipment‐related time per session for image gated proton‐beam therapy including the time for gantry rotation and couch movement, and $T_{p}\left(R\right)$ is the patient‐related time per session.

The patient‐related time per session ($T_{p}\left(R\right)$) includes the times for patient setup (or loading), bone matching, marker matching, and unloading:

(4) $$T_{p}\left(R\right)=T_{l}+T_{b}+T_{m}\left(R\right)+T_{u},$$

where *T_l_* is the patient setup (i.e., loading) time including the time for patient immobilization from when the patient walks into the treatment room, *T_b_* is the time for bone matching, $T_{m}\left(R\right)$ is the time for marker matching, and *T_u_* is the time for patient unloading from the completion of irradiation to when the patient walks out of the treatment room.

The beam delivery time per session for image gated proton‐beam therapy ($T_{\mathrm{BSR}}\left(X,V,R\right)$) is defined as follows:

(5) $$T_{\mathrm{BSR}}\left(X,V,R\right)=T_{\mathrm{BS}}\left(X,V,R\right)+T_{\mathrm{RO}}\left(R\right),$$

where $T_{\mathrm{BS}}\left(X,V,R\right)$ is the beam delivery time per session, as redefined in this study, and $T_{\mathrm{RO}}\left(R\right)$ is the gating operation time.

As it is particularly difficult for patients under 3 years of age to remain still on the couch, general anesthesia or sedation is widely used for safety during treatment.^25^ In the treatment process for pediatric CSI patients with general anesthesia, an additional treatment workflow pertaining to the patient setup and anesthesia machine setup must be included in comparison to the treatment process time for adult patients.

### 2.5 Statistical analysis

Linear approximations and the decision coefficient (*R* ^2^) were obtained to observe the variation between the CTV and beam delivery time per field for each category. The Mann–Whitney's U test was used for all statistical comparisons of *T*_1_ and *T_u_*, which is a common process for all patients regardless of gated irradiation. A *P* < 0.05 was considered statistically significant. All the statistical analyses were performed using JMP Pro 14 (SAS Institute Inc., Cary, NC, USA).

## 3 RESULTS

### 3.1 Patient data

We analyzed the characteristics of the patients in our facility, as listed in Table 1. The patient data (age and sex) were as follows (male:female = 106:46, average age: 55.5 yr, age range: 0–88 yr). We categorized patients based on the disease site. The percentage of patients treated with the gating operation was 59.2%. During the measurement period in this study, there were two cases that required general anesthesia.

### 3.2 Treatment planning

**Figure 3:**
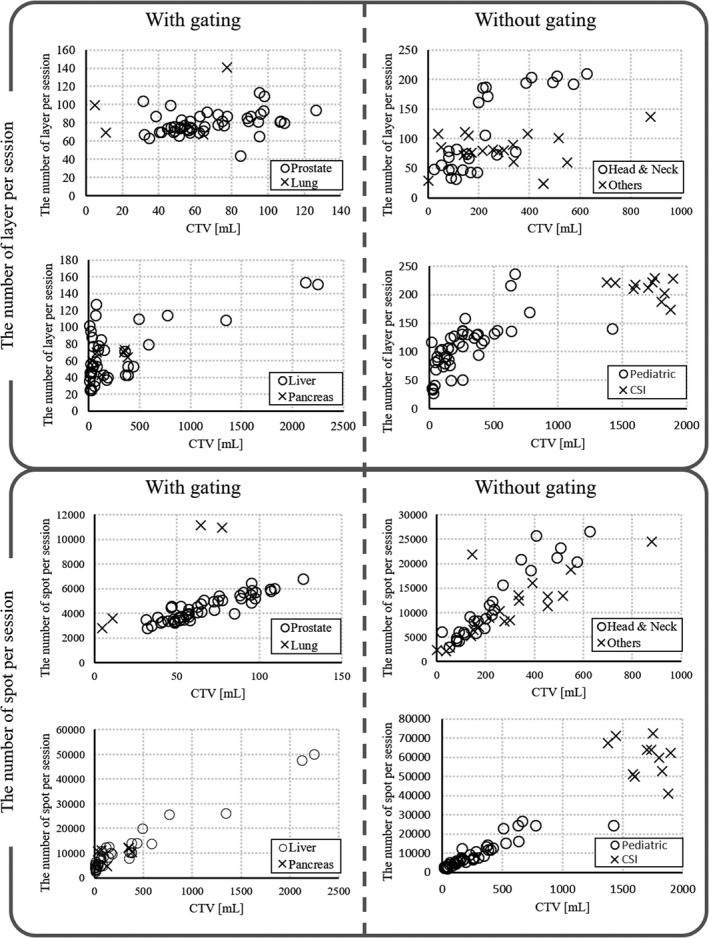
Relationship between the clinical target volume and the numbers of layers and spots per session.

As listed in Table 2, we analyzed all 201 treatment plans including the replan and boost plan. Depending on the patient characteristics, as shown in Fig. 3, the numbers of layers and spots per session increase with the CTV. For two pediatric CSI patients, the entire spine was irradiated with one field.

### 3.3 Treatment process time

We evaluated the treatment process time, which included the duration of each process from when the patient walked into the treatment room to when they walked out. Table 4 lists the measured mean and standard deviation (SD) of the treatment process time from the machine log system for patients of the same category. We evaluated the frequency corresponding to the baseline shift or drift. The frequencies in each category were as follows: Prostate: 16.3%, Liver: 19.7%, Pancreas: 48.8%, and Lung: 45.0%. The higher frequency lead to longer $T_{\mathrm{BSR}}\left(X,V,R\right)$ due to re‐setup.

**Table 4:** Results of the treatment process time for each category. *N* is the number of treatment plans.

Treatment process (min)	Prostate	(n = 48)	Liver	(n = 40)	Pancreas	(n = 6)	Lung	(n = 4)
	Mean ± SD		Mean ± SD		Mean ± SD		Mean ± SD	
With gating								
*T*_1_	4.1 ± 0.5	14.9%	5.4 ± 2.0	16.7%	4.1 ± 0.4	11.4%	5.5 ± 1.2	16.0%
*T_b_*	5.8 ± 1.2	20.8%	6.6 ± 2.6	20.4%	5.7 ± 0.7	16.0%	6.2 ± 1.0	18.3%
$T_{m}\left(R\right)$	3.4 ± 0.9	12.4%	2.8 ± 1.3	8.7%	3.6 ± 1.5	10.3%	2.6 ± 0.3	7.7%
$T_{\mathrm{BSR}}\left(X,V,R\right)$	5.1 ± 1.1	18.4%	8.9 ± 5.8	27.6%	13.4 ± 7.7	37.7%	9.7 ± 3.1	28.4%
*Tu*	3.0 ± 0.7	10.8%	4.3 ± 2.9	13.4%	4.4 ± 0.9	12.5%	4.2 ± 1.7	12.4%
$T_{E}\left(X\right)$	6.3 ± 0.7	22.8%	4.3 ± 1.5	13.3%	4.3 ± 0.5	12.1%	5.8 ± 1.4	17.1%
$T_{\mathrm{total}}\left(X,\;V,\;R\right)$	27.7 ± 2.6	100.0%	32.2 ± 9.8	100.0%	35.5 ± 10.0	100.0%	34.1 ± 2.3	100.0%

Treatment process (min)	Head and neck	(n = 27)	Pediatric	(n = 45)	Others	(*n* = 20)	CSI	(n = 11)
	Mean ± SD		Mean ± SD		Mean ± SD		Mean ± SD	
Without gating								
*T*_1_	4.1 ± 0.8	17.0%	5.4 ± 2.7	20.1%	5.5 ± 1.6	21.1%	9.0 ± 7.1	15.0%
*T_b_*	6.3 ± 1.4	26.2%	7.1 ± 4.1	26.3%	7.7 ± 2.9	29.5%	17.3 ± 3.6	29.0%
$T_{m}\left(R\right)$	NA ± NA	NA	NA ± NA	NA	NA ± NA	NA	NA ± NA	NA
$T_{\mathrm{BSR}}\left(X,V,R\right)$	5.1 ± 3.0	21.2%	4.6 ± 2.0	17.1%	3.8 ± 1.2	14.6%	12.6 ± 1.2	21.0%
*T_u_*	3.1 ± 0.8	12.9%	4.1 ± 2.1	15.2%	4.3 ± 1.9	16.6%	7.5 ± 7.7	12.6%
$T_{E}\left(X\right)$	5.5 ± 1.2	22.7%	5.7 ± 2.7	21.2%	4.7 ± 1.7	18.2%	13.5 ± 3.2	22.5%
$T_{\mathrm{total}}\left(X,\;V,\;R\right)$	24.0 ± 4.5	100.0%	27.0 ± 9.5	100.0%	26.0 ± 4.9	100.0%	59.9 ± 13.9	100.0%

Abbreviation: CSI, craniospinal irradiation.

**Figure 4:**
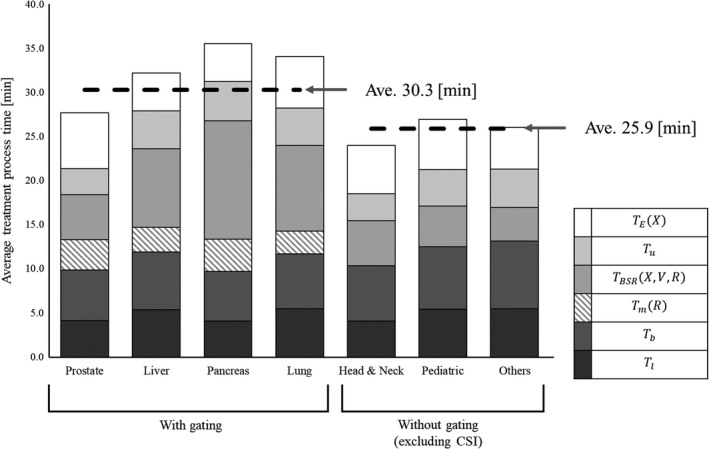
Stacked column chart of the average treatment process time for each category.

The average $T_{\mathrm{total}}\left(X,V,R\right)$ of all the patients during the observation period was 29.9 min, including CSI with anesthesia patients. Figure 4 shows the average treatment process time without gating excluding CSI patients and the average treatment process time with gating. The average $T_{\mathrm{total}}\left(X,V,0\right)$ excluding CSI patients was 25.9 min, whereas the average $T_{\mathrm{total}}\left(X,V,1\right)$ was 30.3 min.

**Figure 5:**
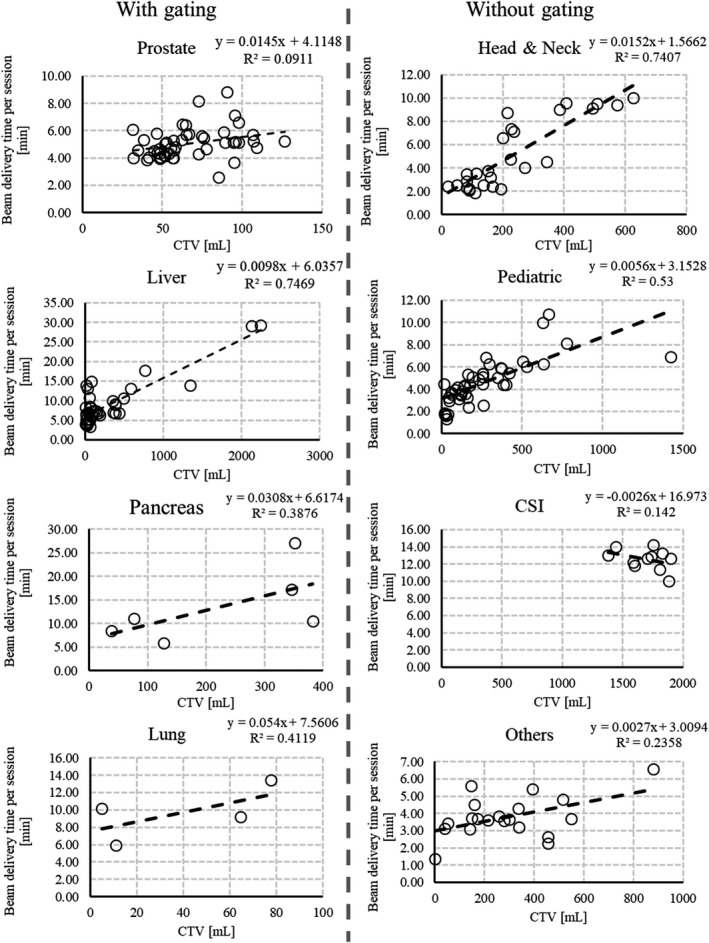
Relationship between the clinical target volume and beam delivery time per session for each category. The dotted line is a linear approximation curve and *R* ^2^ is the decision coefficient.

Figure 5 depicts the relationship between the target volume and beam delivery time per session for each category. The mean and standard deviation of the beam delivery time per session for each category were as follows: Prostate: 5.1 ± 1.1 min, Liver: 8.9 ± 5.8 min, Pancreas: 13.4 ± 7.7 min, Lung: 9.7 ± 3.1 min, Head and Neck: 5.1 ± 3.0 min, Pediatric: 4.6 ± 2.0 min, CSI: 12.6 ± 1.2 min, and others: 3.8 ± 1.2 min. We also fitted with the data by linear approximation. As shown in Fig. 5, the beam delivery time per session increases with the CTV.

For general anesthesia patients, the average general anesthesia process time was 16.3 min for *T_AE_*, 15.4 min for *T_AS_*, and 18.0 min for *T_AF_*. As shown in Table 4, $T_{\mathrm{total}}\left(X,V,R\right)$ is 1 h for CSI patients. Although *T_AS_* and *T_AF_* are included in $T_{\mathrm{total}}\left(X,V,R\right)$, *T_AE_* is not included. As we have only one treatment room, the actual occupancy time of the treatment room requires *T_AE_* to be added to $T_{\mathrm{total}}\left(X,V,R\right)$.

## 4 DISCUSSION

In a previous study, the treatment process time for the passive scattering and spot‐scanning methods were analyzed,^12^, ^13^ and a throughput analysis model for SSPT without the use of the gating function was developed. To the best of our knowledge, this is the first study on the treatment process time with image gated synchrotron‐based proton‐beam therapy using data from the machine log.

Suzuki et al. showed that the daily treatment schedule based on past treatment experience was divided into a fixed time slot of 30 min for a patient with two or three fields per session and 60 min for more fields per session, such as the CSI.^12^, ^13^ Similar to the previous study, our results also showed that in the without‐gating category, the treatment process time for each target (head and neck, pediatric, and others) was at almost the same level.^12^, ^13^ In our result, the average $T_{\mathrm{total}}\left(X,V,1\right)$ was 30.3 min, which is satisfactory for our clinical purposes, and the 30‐min treatment slot can be sufficient, even if the gating function is used. Our study also indicates that it is possible to treat patients using image gated proton‐beam therapy with the same treatment reservation time as that without the gating function.

The average $T_{\mathrm{BSR}}\left(X,V,1\right)$ and $T_{\mathrm{BSR}}\left(X,V,0\right)$ were 7.3 and 4.6 min, respectively. On the other hand, the patient setup time, including *T*_1_, *T_b_*, and *T_m_* (*R*) was 13.9 min with the gating function and 12.0 min without the gating function, excluding CSI. Thus, regardless of the gating function, nearly half the time was spent on the patient setup in the treatment room. The average values of *T_b_*, with and without the gating function excluding CSI, were 6.2 and 7.0 min, respectively. On the other hand, the average *T_b_* for CSI was 17.8 min. This is because the duration for the bone matching process depends on the number of isocenters. Moreover, in the process of bone matching, *T_b_* and its accuracy depend on the skill of the therapist. A reduction in the patient setup time will lead to improved patient throughput. To shorten this time, it is expected that technology for a more efficient setup will be developed, such as automatic positioning developed by utilizing big data gleaned from multiple treatments.

Motion management is important in SSPT. In gating irradiation, the treatment process time required for irradiation needs to be extended compared to the traditional case without gating irradiation. In this study, the mean and SD of $T_{\mathrm{total}}\left(X,V,R\right)$ with the gating function was 30.3 ± 7.4 min. Suzuki et al. demonstrated that the mean and SD of $T_{\mathrm{total}}\left(X,V,R\right)$ without the gating function was 34.4 ± 6.9 min for three fields and 36.9 ± 6.0 min for four including parallel operation, and the treatment reservation time was 30 min for two or three fields per session.^13^ This indicates that the treatment schedule for one session can be a 30‐min slot, even with gating operation. Our study will be beneficial for many institutions that are considering marker‐based gated proton‐beam therapy for moving tumors.

To observe the difference between the CTV and beam delivery time per session, we performed linear approximation. As shown in Fig. 5, in the case without gated irradiation excluding CSI, the beam delivery time per session increased almost linearly as a function of the CTV. On the other hand, in the case with gated irradiation, the beam delivery time per session had considerable variation, depending on the patient‐related factors such as respiration. To shorten $T_{\mathrm{BSR}}\left(X,V,R\right)$, as $T_{\mathrm{BSR}}\left(X,V,R\right)$ depends on the number of layers and spots, when calculating a uniform dose distribution with a proton pencil beam, it may be effective to reduce the number of layers and spots by broadening the proton pencil beam.^26^ Matsuura et al.^27^ developed and evaluated an applicator to broad the Bragg peaks by a mini‐ridge filter for treating superficial moving tumors.

Mizumoto et al.^28^ showed that anesthesia preparation for pediatric patient treatment is beneficial in reducing the treatment time. We performed this preparation before treatment. However, as we have only one treatment room, other patients cannot be treated during the administration or preparation of anesthesia, and this limits our throughput capacity. In this study, we recorded the treatment process for anesthesia patients, for whom anesthesia was administered in the treatment room. To increase the throughput capacity for a gantry facility, the introduction of anesthesia before irradiation and awakening from anesthesia after irradiation by anesthesiologists outside the treatment room can be considered as one of the methods.

The average values of *T*_1_ with and without the gating function excluding CSI patients were 4.7 ± 1.5 min and 5.0 ± 2.2 min (*P* = 0.22), respectively, and those for *T_u_* were 3.7 ± 2.0 min and 3.8 ± 1.8 min (*P* = 0.34), respectively. There was no statistically significant difference between the values with and without the gating function. The average *T*_1_ and *T_u_* for CSI were 9.0 ± 7.1 min and 7.5 ± 7.7 min, respectively, but this difference is because the general anesthesia sessions were included.

As shown in Fig. 2, the time for marker matching does not exist for the case without gating; hence, $T_{m}\left(R\right)$ is the additional time for gating. Despite this, our results for $T_{\mathrm{BSR}}\left(X,V,R\right)$ were within the clinically acceptable range. In addition, $T_{\mathrm{BSR}}\left(X,V,R\right)$ is affected by the operation of the accelerator.^13^ Therefore, in order to improve the gating efficiency, accelerator operation is also important for marker‐based gated proton‐beam therapy. Tsunashima et al. simulated the efficiency of respiratory‐gated‐delivery synchrotron operation for passive scattered proton‐beam therapy.^7^ They concluded that the respiratory gated delivery of synchrotron‐based proton irradiation is feasible and more efficient with a variable magnet excitation cycle pattern.

This study has certain limitations. One is its dependence on patient conditions. Because a patient‐related treatment process for gated irradiation is dependent on the physical and clinical conditions of the patient, such as the state of breathing or peristaltic movement of the intestinal tract, the variation in the treatment process time for a patient‐related treatment process is relatively large compared to that of an equipment‐related treatment process.

Another limitation is the availability of only one treatment room. Our results indicated that the treatment process time depends on the patient setup time. There are certain conditions that cannot be recorded in this study, such as the room switch time. Hence, attention is required in applying the results of this study for multiroom facilities. Moreover, for pediatric CSI with general anesthesia, we need additional time for the anesthesia machine and patient setup compared to the other patients. Additionally, we need a longer setup time for multi‐isocenter treatment plans because *T_b_* increases depending on the number of isocenters. In our results, the average $T_{\mathrm{total}}\left(X,V,R\right)$ for anesthesia patients was 93.2 min (X = 4) and 66.2 min (X = 3). From these results, there is a possibility that the general anesthesia time can be shortened by approximately 20 min. The reduction in the number of fields decreases $T_{\mathrm{total}}\left(X,V,R\right),$ which in turn reduces the anesthetic time for the patient. In our facility, the maximum in‐line length of the field size at the isocenter level is 40 cm. To reduce the number of fields for CSI, a longer in‐line length can be used. However, a field longer than 40 cm might not be realistic.

## 5 CONCLUSIONS

It is important for many proton therapy facilities to evaluate the maximum daily treatment capacity that summation of the treatment process time per session for each patient. For without gated irradiation, treatment process time per session is generally set a 30‐min time slot. On the other hand, it was not clear whether a similar time slot is acceptable even for gated‐spot‐scanning proton‐beam delivery with implanted fiducial marker. In this study, we evaluated the treatment process time for an RGPT system using the data obtained from the machine log files. Our results for the treatment process time demonstrated that SSPT with a gating function can be achieved in approximately 30‐min time slots.

## CONFLICT OF INTEREST

Dr. Shirato reports grants from Hitachi, Ltd. and Shimadzu Corporation during the study. In addition, Dr. Shirato has a licensed patent titled “Moving body pursuit irradiating device and positioning method using this device” and a licensed patent titled “Charged particle beam system” (US 14/524,495). Dr. Umegaki reports grants from Hitachi, Ltd. during the study. In addition, Dr. Umegaki has a licensed patent titled “Charged particle beam system” (US 14/524,495) and a licensed patent titled “Radiotherapy control apparatus and radiotherapy control.” Dr. Shimizu reports grants from Hitachi, Ltd. during the study. In addition, Dr. Shimizu has a licensed patent titled “Charged particle beam system” (US 14/524,495), and a licensed patent titled “Radiotherapy control apparatus and radiotherapy control program” (US9616249). The other authors have no relevant conflicts of interest to disclose.
